# RETRACTED: Awan et al. The Enhanced Cytotoxic and Pro-Apoptotic Effects of Optimized Simvastatin-Loaded Emulsomes on MCF-7 Breast Cancer Cells. *Pharmaceutics* 2020, *12*, 597

**DOI:** 10.3390/pharmaceutics16020191

**Published:** 2024-01-29

**Authors:** Zuhier A. Awan, Usama A. Fahmy, Shaimaa M. Badr-Eldin, Tarek S. Ibrahim, Hani Z. Asfour, Mohammed W. Al-Rabia, Anas Alfarsi, Nabil A. Alhakamy, Wesam H. Abdulaal, Hadeel Al Sadoun, Nawal Helmi, Ahmad O. Noor, Filippo Caraci, Diena M. Almasri, Giuseppe Caruso

**Affiliations:** 1 Department of Clinical Biochemistry, Faculty of Medicine, King Abdulaziz University, Jeddah 21589, Saudi Arabia; zawan@kau.edu.sa; 2 Department of Pharmaceutics, Faculty of Pharmacy, King Abdulaziz University, Jeddah 21589, Saudi Arabia; uahmedkauedu.sa@kau.edu.sa (U.A.F.); smbali@kau.edu.sa (S.M.B.-E.); mr_alfarsi8@hotmail.com (A.A.); nalhakamy@kau.edu.sa (N.A.A.); 3 Advanced Drug Delivery Research Group, Faculty of Pharmacy, King Abdulaziz University, Jeddah 21589, Saudi Arabia; 4 Department of Pharmaceutics and Industrial Pharmacy, Faculty of Pharmacy, Cairo University, Cairo 11562, Egypt; 5 Department of Pharmaceutical Chemistry, Faculty of Pharmacy, King Abdulaziz University, Jeddah 21589, Saudi Arabia; tmabrahem@kau.edu.sa; 6 Department of Medical Microbiology and Parasitology, Faculty of Medicine, King Abdulaziz University, Jeddah 21589, Saudi Arabia; hasfour@kau.edu.sa (H.Z.A.); mwalrabia@kau.edu.sa (M.W.A.-R.); 7 Center of Excellence for Drug Research and Pharmaceutical Industries, King Abdulaziz University, Jeddah 21589, Saudi Arabia; 8 King Fahd Medical Research Center, King Abdulaziz University, Jeddah 21589, Saudi Arabia; 9 Department of Biochemistry, Cancer Metabolism and Epigenetic Unit, Faculty of Science, King Abdulaziz University, Jeddah 21589, Saudi Arabia; whabdulaal@kau.edu.sa; 10 Department of Medical Laboratory Technology, Faculty of Applied Medical Sciences, King Abdulaziz University, Jeddah 21589, Saudi Arabia; hsadounkau.ed.sa@kau.edu.sa; 11 Department of Medical Laboratory Technology, College of Applied Medical Sciences, University of Jeddah, Jeddah 21959, Saudi Arabia; nmhelmi@uj.edu.sa; 12 Department of Biochemistry, College of Sciences, University of Jeddah, Jeddah 21959, Saudi Arabia; 13 Pharmacy Practice Department, Faculty of Pharmacy, King Abdulaziz University, Jeddah 21589, Saudi Arabia; aonoor@kau.edu.sa (A.O.N.); dalmasri@kau.edu.sa (D.M.A.); 14 Oasi Research Institute—IRCCS, Via Conte Ruggero, 73, 94018 Troina, EN, Italy; carafil@hotmail.com; 15 Department of Drug Sciences, University of Catania, 95125 Catania, Italy

The journal retracts the article, “The Enhanced Cytotoxic and Pro-Apoptotic Effects of Optimized Simvastatin-Loaded Emulsomes on MCF-7 Breast Cancer Cells” [1], cited above.

Following publication, concerns were brought to the attention of the publisher regarding a range of image issues.

Adhering to our complaint’s procedure, an investigation was conducted by the Editorial Office and the Editorial Board that confirmed the presence of Figure 4 in two prior publications [2,3]. Furthermore, Figure 8 (panel B) was also confirmed to be similar to some images from other publications [4,5,6,7,8,9], and Figure 7A,B is similar to the images from another paper [7].

While the authors fully cooperated with the Editorial Office during the investigation, they were unable to satisfactorily explain the similarity of the figures presenting different experimental conditions, nor meet the required quality standards of raw images in order to consider a correction, as per the journals original image requirements policy (https://www.mdpi.com/journal/pharmaceutics/instruc-tions#oriimages). As a result, the Editorial Board and EIC were unable to confirm the reliability of the findings and subsequently decided to retract this paper.

This retraction was approved by the Editor-in-Chief of the journal *Pharmaceutics*.

The authors did not agree to this retraction.

## References

[1] Awan Z.A., Fahmy U.A., Badr-Eldin S.M., Ibrahim T.S., Asfour H.Z., Al-Rabia M.W., Alfarsi A., Alhakamy N.A., Abdulaal W.H., Al Sadoun H. (2020). RETRACTED: The Enhanced Cytotoxic and Pro-Apoptotic Effects of Optimized Simvastatin-Loaded Emulsomes on MCF-7 Breast Cancer Cells. Pharmaceutics.

[2] Fahmy U.A., Badr-Eldin S.M., Ahmed O.A.A., Aldawsari H.M., Tima S., Asfour H.Z., Al-Rabia M.W., Negm A.A., Sultan M.H., Madkhali O.A.A. (2020). Intranasal Niosomal In Situ Gel as a Promising Approach for Enhancing Flibanserin Bioavailability and Brain Delivery: In Vitro Optimization and Ex Vivo/In Vivo Evaluation. Pharmaceutics.

[3] Fahmy U.A., Alaofi A.L., Awan Z.A., Alqarni H.M., Alhakamy N.A. (2023). Retraction Note: Optimization of Thymoquinone-Loaded Coconut Oil Nanostructured Lipid Carriers for the Management of Ethanol-Induced Ulcer. AAPS PharmSciTech.

[4] Aldawsari H.M., Ahmed O.A.A., Alhakamy N.A., Neamatallah T., Fahmy U.A., Badr-Eldin S.M. (2021). Lipidic Nano-Sized Emulsomes Potentiates the Cytotoxic and Apoptotic Effects of Raloxifene Hydrochloride in MCF-7 Human Breast Cancer Cells: Factorial Analysis and In Vitro Anti-Tumor Activity Assessment. Pharmaceutics.

[5] Alhakamy N.A., Badr-Eldin S.M., Ahmed O.A.A., Asfour H.Z., Aldawsari H.M., Algandaby M.M., Eid B.G., Abdel-Naim A.B., Awan Z.A., Alghaith A.F. (2020). Piceatannol-Loaded Emulsomes Exhibit Enhanced Cytostatic and Apoptotic Activities in Colon Cancer Cells. Antioxidants.

[6] Alhakamy N.A., Badr-Eldin S.M., Aldawsari H.M., Alfarsi A., Neamatallah T., Okbazghi S.Z., Fahmy U.A., Ahmad O.A.A., Eid B.G., Mahdi W.A. (2023). Retraction Note: Fluvastatin-Loaded Emulsomes Exhibit Improved Cytotoxic and Apoptosis in Prostate Cancer Cells. AAPS PharmSciTech.

[7] Badr-Eldin S.M., Aldawsari H.M., Ahmed O.A., Alhakamy N.A., Neamatallah T., Okbazghi S.Z., Fahmy U.A. (2021). Optimized semisolid self-nanoemulsifying system based on glyceryl behenate: A potential nanoplatform for enhancing antitumor activity of raloxifene hydrochloride in MCF-7 human breast cancer cells. Int. J. Pharm..

[8] Alhakamy N.A., Badr-Eldin S.M., Fahmy U.A., Alruwaili N.K., Awan Z.A., Caruso G., Alfaleh M.A., Alaofi A.L., Arif F.O., Ahmed O.A.A. (2020). Thymoquinone-Loaded Soy-Phospholipid-Based Phytosomes Exhibit Anticancer Potential against Human Lung Cancer Cells. Pharmaceutics.

[9] Alhakamy N.A., Fahmy U.A., Badr-Eldin S.M., Ahmed O.A.A., Asfour H.Z., Aldawsari H.M., Algandaby M.M., Eid B.G., Abdel-Naim A.B., Awan Z.A. (2020). Optimized Icariin Phytosomes Exhibit Enhanced Cytotoxicity and Apoptosis-Inducing Activities in Ovarian Cancer Cells. Pharmaceutics.

